# A Deeper Look into Type 1 Diabetes – Imaging Immune Responses during Onset of Disease

**DOI:** 10.3389/fimmu.2016.00313

**Published:** 2016-08-15

**Authors:** Gustaf Christoffersson, Matthias G. von Herrath

**Affiliations:** ^1^Type 1 Diabetes Center, La Jolla Institute for Allergy and Immunology, La Jolla, CA, USA; ^2^Novo Nordisk Diabetes Research and Development Center, Seattle, WA, USA

**Keywords:** type 1 diabetes, autoimmunity, imaging, microscopy, confocal, intravital imaging

## Abstract

Cytotoxic T lymphocytes execute the killing of insulin-producing beta cells during onset of type 1 diabetes mellitus (T1D). The research community has come far in dissecting the major events in the development of this disease, but still the trigger and high-resolved information of the immunological events leading up to beta cell loss are missing. During the past decades, intravital imaging of immune responses has led to significant scientific breakthroughs in diverse models of disease, including T1D. Dynamic imaging of immune cells at the pancreatic islets during T1D onset has been made possible through the development of both advanced microscopes, and animal models that allow long-term immobilization of the pancreas. The use of these modalities has revealed a milling microenvironment at the pancreatic islets during disease onset with a plethora of active players. Clues to answering the remaining questions in this disease may lie in intravital imaging, including how key immune cells traffic to and from the pancreas, and how cells interact at this target tissue. This review highlights and discusses recent studies, models, and techniques focused to understand the immune responses during T1D onset through intravital imaging.

## Introduction

Although type 1 diabetes (T1D) has been studied in great detail during the past century, we still remain oblivious about many steps in the autoimmune process leading up to near complete beta cell destruction and hyperglycemia. Many outstanding questions remain on the events that take place at the pancreatic islets during disease onset: how do the immune cells gain access to the islets? How do immune cells traffic to the pancreas? How do the different immune cell subsets behave and interact with one another at the islets and in draining lymph nodes? How do the immune cells respond to therapeutic intervention to prevent or cure autoimmune diabetes?

At the center of the disease lie the islets of Langerhans which harbor the insulin-producing beta cells inside miniature (100–300 μm) endocrine organs scattered throughout the pancreas. The immune reaction in T1D is focused to these tiny spheroids, and the rest of the pancreas stays rather unaffected by the immune attack. The scale and dispersion of the affected tissue and focal inflammation in combination with the retroperitoneal position of the pancreas pose great challenges for intravital imaging.

Static imaging (e.g., tissue sections) or flow cytometry will identify differences in functional activities if they are large, such as the differential expression of a known protein. In instances where these more profound differences are present between cellular subsets, these modalities may be well adequate for analyzing such cells. However, when differences are more subtle, e.g., only apparent through the differential behavior of a group of cells, real-time imaging would be the modality to distinguish this.

There is great interest in the development of imaging techniques for islets of Langerhans, both experimental and clinical. Using imaging as a diagnostic tool for following changes in beta cell mass or assessing islet inflammation in patients with recent-onset T1D may prove very helpful in early detection and understanding of disease kinetics. As an experimental tool, imaging immune responses in the pancreas and related lymphatic organs has already proven instrumental for understanding of the autoimmune events leading up to beta cell loss.

High-resolved real-time imaging of the pancreas has not yet been broadly applied to study a wider range of immune cells and events in T1D. This focus of this review is to highlight and discuss recent studies, models, and techniques focused to understand the immune responses during T1D onset through intravital imaging of different modalities.

## Imaging Modalities

A dream for many immunologists is to be able to follow immune cells through the body using an imaging modality that does not disturb normal physiology/pathophysiology and that can be used to perform longitudinal studies in the same study subject. Even though it sounds like an unobtainable fantasy, a few methods do come close to fulfilling it; bioluminescence imaging (1), positron emission tomography (PET) (2), single-photon emission computed tomography (SPECT) (3), X-ray computed tomography (CT), and magnetic resonance imaging (MRI) (4) have some of these features. However, for more high-resolved imaging, more invasive and less clinically applicable methods, such as intravital microscopy, are required.

### Bioluminescence Imaging

Cells producing bioluminescence will emit light that penetrates far through tissue, making non-invasive imaging possible. However, luciferase needs to be transgenically expressed in these cells, and a secondary imaging modality (e.g., visible light camera, X-ray CT, MRI) is required for anatomical information. Bioluminescence has been utilized in the field of diabetes research by inserting the gene encoding luciferase under control by the mouse insulin promoter (MIP) (5) enabling non-invasive longitudinal quantification of beta cell mass in the mouse (6). Similarly, bioluminescence imaging can also be used for more detailed functional assessment of beta cell biology, for example, the technique has been used in pancreatic islet transplantation research, for assessing the level of hypoxia in the engraftment process by expressing luciferase under the control of hypoxia response elements (7). With bioluminescence imaging, there are limitations to the extent of multiplexing that can be achieved, and in most current devices only one single channel of information is collected. Even though this modality supports cellular resolution, the above-mentioned aspects limit the use for this modality in more detailed immunological research, and have as such not been exploited to a larger extent in autoimmune research. Some recent work may point to where this modality could be used in inflammation research: a luciferace-based assay was used where the interactions of two different chemokine receptors with beta-arrestin 2, and their activation of signaling can be simultaneously assessed in living cells (8), and the trafficking of transgenic T cells (9) and luciferase-transfected dendritic cells (10) in the whole living animal.

### Radiological Techniques

The anatomical location of the pancreas makes it difficult to access for a number of imaging modalities. However, radiological techniques are in general not to any greater extent limited to tissue diffraction (light-based approaches) or direct access to the organ of interest (direct imaging approaches). Techniques, such as MRI and CT, are widely used in clinics around the world for a variety of diagnostic purposes, and are, thus, attractive modalities also from a translational perspective. The advent of a robust method for assessing islet inflammation using these would provide a bridge from bench to bedside, and offer a way of early T1D diagnosis and precise disease assessment.

The size and distribution of pancreatic islets puts a high demand on spatial resolution for imaging. MRI can achieve a resolution on the 0.1-mm scale, just enough to resolve islets in the pancreas. However, the modality is put to better use in functional imaging, targeting morphological and physiological changes in the pancreas during onset of T1D, such as microvascular changes in flow, volume, and permeability (11, 12). Specific imaging of insulitis has also been successfully explored using MRI. Gaglia and colleagues (13) infused dextran-coated magnetic nanoparticles into healthy and T1D subjects. These nanoparticles are readily phagocytosed by macrophages, and are negative T2-weighted contrast agents enabling the visualization of areas with a high abundance of macrophages, such as inflamed pancreatic islets. Non-invasive imaging of insulitis showed a great heterogeneity among the subjects, and also confirmed previous data in showing that the total pancreas volume is lower in T1D subjects (13).

Magnetic resonance imaging has also been used for the visualization of islet grafts following transplantation. Here, the possibility of *ex vivo* labeling the islets for increased detection was proven feasible (14). When transplanted in larger clusters, islet grafts can be imaged without the use of contrast-enhancing agents, as shown in intramuscular transplantation (15).

For a more in-depth assessment immune cell trafficking using MRI, *ex vivo* labeling of immune cells with iron oxide- or ^19^F-based probes is emerging as a powerful way of non-invasively imaging leukocyte movement at the whole-animal level (4). It has, to our knowledge, yet to be tested in a T1D setting, but could provide important information on trafficking of effectors and suppressors to, from, and within the pancreas.

Computed tomography is routinely used for imaging retroperitoneal organs, such as the pancreas. However, the spatial resolution of the modality is too poor to be able to resolve scattered pancreatic islets. CT has, thus, not been employed to a larger extent as a solitary imaging modality in imaging for assessing T1D progression in the pancreas. CT has been used for imaging grafts following whole-pancreas transplantation for assessing vascularity, thrombosis, steatosis, and lymphocyte infiltration. These less-detailed points of information help in assessing engraftment, but are still too blunt to assess T1D immunology. However, with CT as an anatomic delineator in combination with a nuclear medicine technique, such as PET or SPECT, an increased level of precision can be gained, and an increased amount of information can be gathered. Much effort has been put into finding beta cell-specific tracers for use in PET/SPECT that would provide a way of quantitatively assessing beta cell mass in humans. Radiolabeled compounds targeting beta cells via insulin granules [e.g., dithizone (16)], ATP-sensitive potassium channels [e.g., glibenclamide (17)], vesicular monoamine transporter type 2 [VMAT2, e.g., dihydrotetrabenazine (18)], the GLP-1-receptor [e.g., exendin-4 (19, 20)], and agents targeting beta cell metabolism [e.g., fluorodeoxyglucose (2, 21)] are among the most studied. An accurate method could provide a way of not only longitudinally studying beta cell decay in humans, but also provide a method for early T1D diagnosis.

Nuclear medicine methods can also be used for detecting and following insulitis. *Ex vivo* labeling of lymphocytes can be achieved through the use of agents, such as [^111^In]oxine (22), [^111^In]tropolonate (23), or [^99m^Tc]hexamethylpropyleneamine oxime (24). However, results from both rodent and human studies were disappointing, where very little signal from the transferred lymphocytes were found in the pancreas. Most labeled cells ended up in secondary lymphoid organs, most likely because these sets of polyclonal lymphocytes were not activated and too diverse in their specificities, leading to very few cells finding their way to the islets in the pancreas (25). A more promising approach is the radiolabeling of molecules targeting immune cells *in vivo*. One example is the cytokine interleukin-2 (IL-2). Activated T cells infiltrating the pancreatic islets during T1D will express high levels of the IL-2 receptor and thereby bind and internalize large amounts of IL-2. Thereby, the infusion of [^123^I] labeled IL-2 permitted the visualization of progressing insulitis in non-obese diabetic (NOD) mice (26). For increased flexibility and specificity, moving to radiolabeled antibodies enables the tracking of specific immune cell subsets. The Fc-receptor-binding aspects of polyclonal IgG’s can be utilized if a less-specific staining is enough (27), or the use of, for example, a non-Fc-binding anti-CD3 antibody to specifically label T cells (28). A problem common for the last few modalities above is the low signal-to-noise ratio emanating from the fraction of circulating unbound antibodies/protein, and the relatively low number of T cells present at the islets in human T1D. If these hurdles can be overcome, nuclear medicine methods could provide high cellular specificity in research and diagnosis of T1D.

### Light Microscopy

The simplest and most straightforward way of imaging both static tissue samples and dynamic events in live organisms is by merely using visible light to illuminate the object and combining this with a means of magnifying the area. In the sixteenth century, the Dutch spectacle makers Janssen constructed the first compound microscope. This was the start of a now 400-year journey deeper into the physiology of all living things, culminating in the 2014 Nobel prize (Betzig, Hell, and Moerner) acknowledging the development of super-resolution microscopes, which defy previous dogmas of the limit of resolution in ingenious ways.

In the area of microcirculation and inflammation, a great body of knowledge has been gained during the late nineteenth and early twentieth centuries by relatively rudimentary microscope setups where, for example, the wings of bats or the tongues of frogs were studied through transmitted-light microscopes. With the advent of epifluorescence microscopy, scientists were no longer limited to translucent tissues for imaging. Using reagents capable of emitting fluorescent light, also structures and events in more dense organs could be imaged, such as blood flow using fluorescently tagged dextran. The major leap in the field came with the confocal microscope (29). With this technique, the light returning from the epi-illuminated tissue is confined by the use of a pinhole or a slit, limiting the collection of photons to only a narrow focal plane. Apart from decreasing noise in imaging, the optical sectioning feature of confocal microscopy enabled three-dimensional reconstructions of the collected data, which greatly aids in studying cell-to-cell communications in a tissue context. With the development of multiphoton microscopy in the 1990s (30), a couple of the major drawbacks with confocal laser scanning microscopy were avoided; namely high phototoxicity and poor tissue penetration (~100 μm). By using a pulsed laser that emits light in the far-red to infra-red spectrum (>690 nm), normal fluorophores expressed or delivered to the tissue will only emit light when they absorb two photons on a very short time-scale (10^−15^ s) in multiphoton microscopy. The probability of this occurring in another spot than the focal point is very low, limiting excitation of the tissue to a very small area. The longer laser wavelengths used in multiphoton microscopes also reduces the amount of energy transferred to the tissue, decreasing the risk of phototoxicity, and increases the penetration depth (~500 μm) due to decreased light-scattering effects in tissue. The increased penetration depth offered by multiphoton microscopy is a great advantage to visible-light confocal microscopy when imaging islets in the pancreas. With a normal confocal microscope, the microscopist is limited to islets situated at the very surface of the pancreas, limiting the selection of tissue to be studied. Increasing the penetration depth by only a few hundred micrometers will greatly increase the numbers of islets that are reachable for the microscope. Other features of multiphoton excitation are second (two-photon excitation) and third (three-photon excitation) harmonic generations. In these, multiple photons interact with tissue constituents and create a resulting photon of two or three times the initial energy, respectively. This enables label-free imaging of collagen and myosin fibers [second harmonic generation (31)], and lipid layers and myelin fibers [third harmonic generation (32)]. In combination with the collection of reflected laser light, multiple channels of morphologic information of the tissue can be collected without the use of fluorescent dyes.

A few modalities have emerged recently that expand the possibility to collect more information from intravital microscopy preparations. Coherent anti-Stokes Raman scattering (CARS) uses two laser beams to investigate the energy gap between two vibrational levels of a studied molecule. This modality has proven useful for imaging myelin fibers and lipid layers (33–35). Fluorescence lifetime imaging (FLIM) interrogates the time a molecule spends in an excited state, and can give quantitative information on parameters, such as ion concentrations, pH, metabolic states, and oxygen levels (36–38).

## Live Cell and Intravital Imaging

### High-Resolution Intravital Imaging of Immune Responses

The increased accessibility to laser scanning confocal microscopes in the 2000s spurred a surge in new discoveries and insights into immunological events. This was also fueled by the concurrent development of imaging tools, such as fluorescent proteins, the development of which was awarded the Nobel prize in 2008 (Chalfie, Shimomura, and Tsien). Mouse models where certain immune cell subsets express fluorescent reporter genes have led to discoveries that were unlikely to occur without the use of high-resolved imaging. One example is the widely used Cx3cr1^GFP^ reporter mouse, where predominantly monocytes and macrophages are tagged with the fluorescent protein (39). This reporter mouse has revealed many secrets held by these myeloid cells, including a previously unappreciated vast distribution of resident macrophages (40), and the subsetting and exposure of dual functions of monocytes (41, 42).

The most common organs to image in immunological questions are lymphoid organs, such as draining lymph nodes and the spleen. These organs are not only highly relevant sites for the integration of immunological signals but are also relatively uncomplicated to image. They require minimal surgery [e.g., a skin flap for inguinal or cervical lymph node (43), or the ear (44)], and can even be imaged *ex vivo* as explants in heated chambers with satisfying results [e.g., vibratome-cut spleen (45)]. The introduction of the multiphoton microscope and its improved imaging depth further increased the ability to interrogate questions in lymphatic organs. Intravital microscopy in the field of immunology has not just put images on processes that were already know, but has proved to be a very powerful tool to unveil interactions and movements that were impossible to uncover without direct microscopy. We now know that leukocytes crawl within vasculature (42, 46), that intraperitoneal macrophages invade the damaged liver (47), that there is a very intricate interplay between B cells and T helper cells in germinal centers (48), and the swarming behavior of innate leukocytes when tissue is damaged (44, 49).

### Intravital Imaging of the Pancreas

Due to its retroperitoneal location and its fragility *in vivo* imaging of the pancreas poses difficulties on the operator performing the imaging. In particular, the scattered distribution of the islets of Langerhans puts high demands on the tools used for imaging for allowing both deep tissue imaging and the ability to keep a single islet in focus for extended periods of imaging.

When performing intravital microscopy imaging on small animals, normal physiology will in one way or another be disturbed. The surgery involved will provoke some inflammation and fluid leakage. Materials used to immobilize the animal or organ may disrupt blood flow or function as heat sinks if made of a conductive material. The sedated state of the animal, including off-target effects from the anesthetics, will render issues with temperature regulation. Also laser illumination of the sample can cause heating of the tissue or other forms of phototoxicity, such as cell damage due to the generation of free oxygen radicals. Measures should, thus, be taken to support normal physiology to as large extent as possible, from heating pads under the animal to the monitoring of blood pressure, and monitoring of tissue perfusion in the organ studied to be able to achieve reproducible and true results.

All of the parameters mentioned above need to be taken into account when performing imaging of the live pancreas or islets transplanted elsewhere. A number of different methods for imaging islets in the living rodent have been published over the years (Table 1). Common to all methods is the ambition to create a state where the tissue is as isolated as possible from respiratory and cardiac movements, while still maintaining undisturbed blood perfusion, moisture, and temperature.

**Table 1:** **Methods for intravital microscopy of immune responses in pancreatic islets**.

***In situ* in the pancreas**	
In peritoneum	Hellerstrom and Hellman (50), McCuskey and Chapman (51), and Rooth et al. (52)
Exteriorized on pedestal	Martinic and von Herrath (53) and Coppieters et al. (54)
Suction ring	Lindsay et al. (55)
**Transplanted**	
Dorsal skin fold	Menger et al. (56)
Striated muscle	Christoffersson et al. (15)
Anterior chamber of the eye	Speier et al. (57)
Under the kidney capsule	Bertera et al. (58)
Greater omentum	Espes et al. (59)
**Live explants**	
Isolated islets	Friedman et al. (60)
Sectioned pancreas	Marciniak et al. (61)

Intravital fluorescence imaging of the physiology of the pancreas and the islets of Langerhans has been performed since the 1960s (50) when Hellerstrom and Hellman did their intravital observations on the islet blood flow *in situ*. The early fluorescence microscopy studies by Rooth and colleagues in the 1980s showed the usefulness of direct microscopy of dynamic events in pancreas physiology (52), as their observations of a “stop-and-go” blood flow in islets following norepinephrine infusion explained why two other groups had obtained opposing results (62, 63). Investigations of, for example, the vascular network, blood flow, and leukocyte recruitment do not require advanced microscopes (i.e., wide-field trans/epi-illumination microscopes), but renders the researcher to be limited to islets that are superficially situated and easily distinguished. More recently, Nyman et al. (64) used the three-dimensional power of the confocal microscope to answer the long debated question of blood flow direction in pancreatic islets of the mouse. They found two distinct blood flow patterns: one “inner-to-outer” and one “top-to-bottom” direction of flow (64).

**Figure 1:**
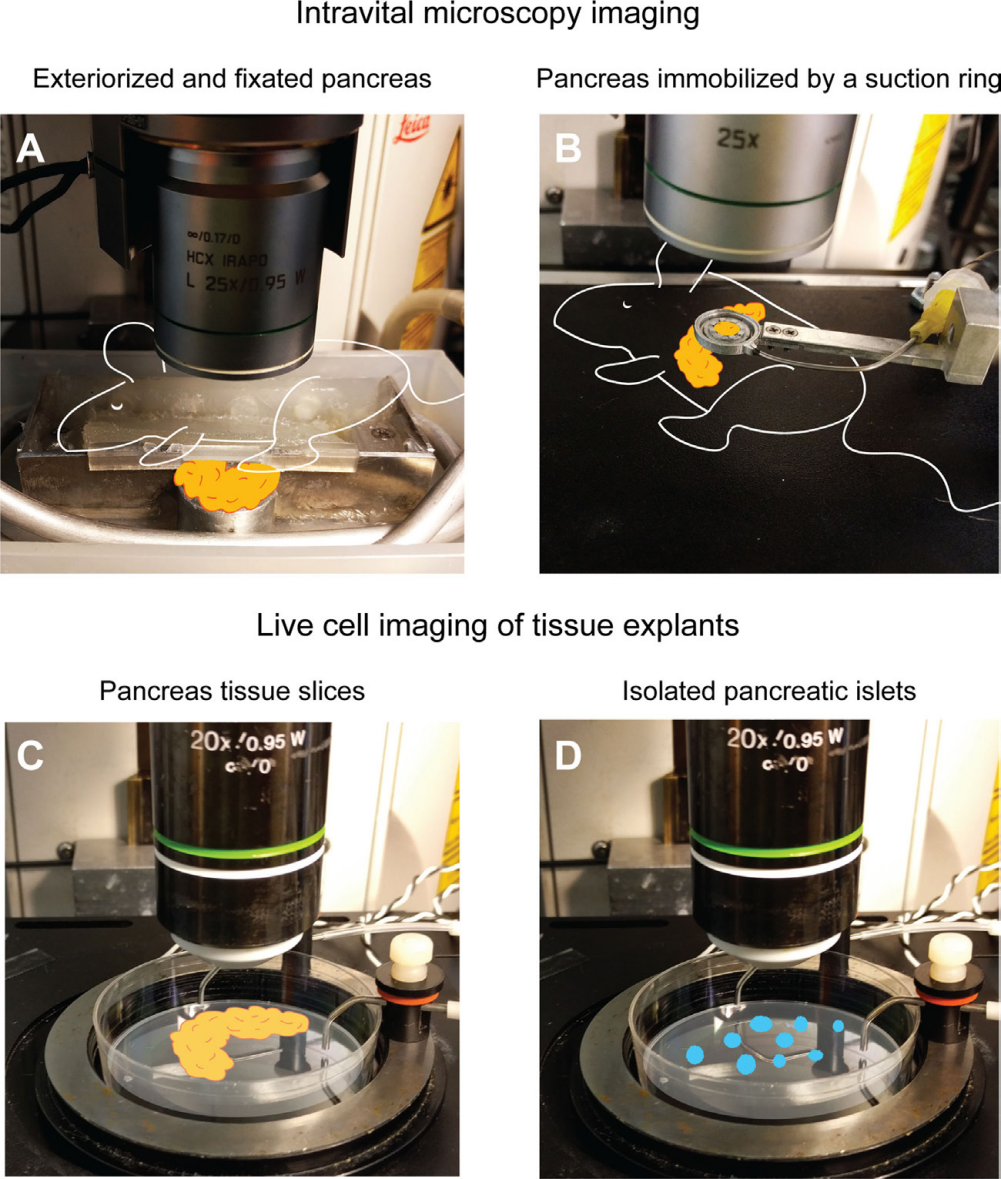
**Examples of intravital and live cell imaging methods for T1D research**. **(A)** The pancreas can be exteriorized and fixed to a viewing pedestal for motion-artifact free imaging. In this particular setup, the mouse and pancreas are kept warm by body-temperature water circulating in the metal tubing. The pancreas is kept moist by being submerged in physiologic saline buffer. For more details, see Ref. (54). The suction ring pictured in **(B)** allows for the pancreas to remain in the abdominal cavity while immobilizing a small part of the tail region to a circular coverslip for intravital imaging. For more details, see Ref. (55, 66). If more advanced manipulation of the tissue is needed or intravital microscopy is otherwise impossible, thick, live sections of the pancreas in a warm imaging chamber **(C)** can be an alternative to *in vivo* methods. For details, see Ref. (67). If only pancreatic islets are available or required for the research questions, immobilization in agarose gel and placement in a warm imaging chamber **(D)** can provide live cell imaging. For details, see Ref. (55).

Due to anatomical and microscopy technical issues, it was not until 2010 detailed intravital observations of the immune response during onset of T1D were first made. Coppieters et al. had developed a model where the pancreata of mice were exteriorized and kept warm and moist on a viewing pedestal for confocal/multiphoton imaging (53, 54). Since, in this model, the pancreas was exteriorized and attached to a foundation, it was isolated from respiratory artifacts from the mouse (Figure 1A). In comparison to former *in situ* imaging models, this allowed for imaging a larger area of the pancreas (albeit biased to the tail region of the organ). In combination with multiphoton imaging and fluorescent reporter mice, this model revealed new insights into the dynamics of cytotoxic T lymphocyte (CTL) trafficking and actions in the pancreas during beta cell killing (65).

A leap forward in intravital microscopy imaging of several different organs of the mouse was made with the development of a suction window where the organ of interest is kept in a stable position under a coverslip by some gentle vacuum [Figure 1B (66)]. The method was first developed for imaging the lung, but it has proven useful for a wide range of other organs, including the pancreas (60). The advantages that the suction window offered were an increased stability during imaging, as a small part of the pancreas is held steady by the vacuum, and that the pancreas can remain in its native location in the peritoneal cavity. Access to the tissue only requires an incision in the left peritoneal wall, small enough to fit the suction device, resulting in minimized fluid loss and an increased ability to maintain euthermia in the organ. The device is also small, and can be 3D-printed in a plastic material, offering customizability to a very low price. Some computer-aided design (CAD) drawings of different ring designs have been deposited online for free download from the University of California, San Francisco, CA, USA (http://biomicroscopy.ucsf.edu).

The above-mentioned methods do, however, not allow for longitudinal imaging of the same pancreas in the same animal. The surgical procedures surrounding these imaging techniques require the experiments to be terminal. A solution could be the surgical implantation of an abdominal imaging window (68). To our knowledge, this has yet to be proven successful in imaging the progress of T1D in pancreatic islets *in situ*, but implanted body windows have provided useful longitudinal data from other abdominal organs (69) and tumors (70).

A sidetrack from intravital imaging is live cell imaging of tissue explants. Even though this is a step far from the *in vivo* situation where the tissue lacks vital components, such as blood flow, influx of immune cells, lymphatic drainage etc., it might still be useful for some questions in immunology. Imaging of T cell motility in isolated islets from NOD mice placed in an agarose gel (Figure 1D) was successfully performed by Lindsay et al. (55). This group also performed intravital imaging of the same events to validate the method by correlating *in vivo* migration data to that from explanted islets. Arguments for performing this type of explant imaging as compared to intravital imaging are the increased number of islets that can be imaged per pancreas used, that lower-intensity fluorescent reporters, such as biosensors, can be used, and that an increased level of manipulation of the tissue can be introduced. Drawbacks include the fact that only immune events within the islets are recorded, when much of the dynamics of T cell migration and interactions may occur in the islet periphery. The isolation of islets also induces stress, and limits the study to fairly intact islets, and not islets in more advanced stages of destruction as these would not survive tissue digestion protocols.

Even though the method has not yet been proven for immunological questions, the use of thick, live slices of pancreata may be a way of performing live cell imaging when an increased level of manipulation is required (Figure 1C), or when the tissue comes from large animals, such as pigs and non-human primates or even donated human pancreata. Marciniak and colleagues (67) embedded pancreatic tissue in agarose gel and then used a vibratome to cut thick slices (150 μm) of the tissue in a minimally disruptive way. As a proof of concept for the usefulness of the model in islet physiology, the group performed calcium imaging of beta cells in the pancreas slices using a genetically encoded calcium indicator (67). A model such as this also opens up for more advanced manipulation of the tissue, including that related to immune cells, such as patch-clamping and other electrophysiological and neurophysiological experimentation.

**Figure 2:**
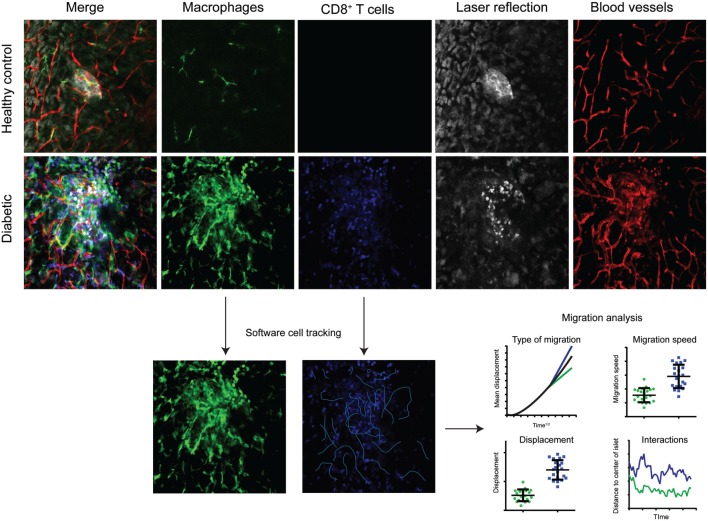
**Multicolor intravital confocal imaging of the pancreas**. Examples of still-frames from intravital microscopy imaging of the pancreas in healthy control mice (top row) and diabetic (LCMV.RIP-GP) mice (middle and lower rows). Macrophages are visualized by their expression of GFP at the CX_3_CR1 locus (green). Adoptively transferred antigen-specific P14 T cells express the fluorescent DsRed protein. Pancreatic islets and parenchyma are visualized by laser reflection. Notice the difference in reflective signal as the beta cells are killed and enter apoptosis. Blood vessels are visualized by the injection of a fluorescently tagged anti-CD31 antibody. Four-dimensional data (three spatial dimensions plus time) is collected and used for software tracking of cell movements and the interactions between T cells and beta cells, and T cells and antigen-presenting cells. The type of migration can be distinguished by plotting mean displacement to the square root of time. The three lines represent random/Brownian movement (black), confined movement (green), and directional movement (blue).

Common to most intravital microscopy modalities are the large quantities of data that are gathered for each experiment. Confocal and multiphoton microscopes provide four-dimensional information (three spatial dimensions plus time) (Figure 2). This in combination with multicolor imaging over extended periods of time renders several gigabytes of data per day in a microscopy-focused lab. Good routines for data storage and handling are important. Important are also the softwares used for analyzing the recorded images. Well-written scripts can simplify the extraction of valuable data from 4D movies. A number of high-performing alternatives exist, several with a full- or semi-open source profile for researchers to write and share their own scripts, macros, and add-ons. A recent example is the merge of flow cytometry-type of data visualization with imaging software (71).

### Intravital Imaging of Transplanted Islets

A way of circumventing the difficulties involved in direct intravital microscopy of the pancreas has been to isolate pancreatic islets and to syngeneically transplant them elsewhere in the body where imaging could more readily be performed. Examining islet physiology by imaging them at a different site than the pancreas was pioneered by Menger and colleagues in the 1980s (56). This group investigated the regulation of microvascular blood flow and angiogenic capacities of the islets transplanted into a dorsal skin fold (72). Others have imaged the engraftment and revascularization of islets in the context of curative transplantations (15), and used islets transplanted to muscle as a model for studying non-tumoral angiogenesis (73). Pancreatic islets and pancreatic tissue have been transplanted to the anterior chamber of the eye for experimental studies for a long time (74). However, it was not until Speier and colleagues combined the technique of inserting islets into the eye of rodents with advanced intravital microscopy that a powerful tool for studying the islet engraftment process and subsequently beta cell biology in an *in vivo* setting emerged (75). The main advantage of this model is the non-invasive way of imaging islets through the eye of the animal. The cornea acts like a natural body window and allows for longitudinal imaging of the same islet over the course of the animal’s life. The eye has also been proven not to be a completely immune privileged site when it comes to allorejection (76) and autoimmune destruction (77) of the beta cells. Another method that also provided longitudinal imaging of islets was the body-window method described by Bertera et al. (58). The authors transplanted fluorescence transgenic islets under the kidney capsule of mice and inserted a body window to be able to view the islets repeatedly over time.

Even though the methods described above provide elegant solutions to imaging the immune responses at islets during onset of T1D, careful consideration of how the microenvironment has changed has to be done before extrapolation of the results can be made to be true also for the pancreas. Differences in migration substrates for the immune cells (78), lymphatic drainage (79), or populations of resident leukocytes (80) can all affect the nature of the immune response targeted to the beta cells.

## Interrogations in T1D Requiring Intravital Imaging

Many questions remain unanswered in autoimmune diabetes and great efforts are under way to provide new insights into this enigmatic disease. Prospective cohorts, genetic analyses, epidemiologic datamining, and other large projects are set to contribute some of these, but to get to the core of the causative events of T1D, a deep look into the events that take place at the islets of Langerhans before and during beta cell destruction might be what holds the key to a cure.

With the work performed by Coppieters et al. (54, 65), we now have a much better view of some of the T cell trafficking events that take place during onset of experimental autoimmune diabetes. With the use of a mouse model [LCMV.RIP-GP (81)] where a well-defined autoantigen is transgenically presented on the surface of beta cells, and fluorescent antigen-specific CD8^+^ T cells (P14), the antigen-governed immune response at the islets could be tracked. These studies showed that CTL gain access to the pancreas via extravasation through postcapillary venules and freely migrate through the pancreas in a random-walk fashion. These observations suggest that there are no apparent barriers for the CTLs in the mouse pancreas as has been suggested in other studies (82), but they move freely in and out of the islets with no time-lag at the islet–exocrine interface.

Tracking of antigen-specific T cells in intravital recordings is readily done by using an artificial and transgenic system, such as the RIP-GP/P14/Smarta model mentioned above, or the RIP-mOVA/OT-I/OT-II model (83). The adoptive transfer of fluorescent CD4^+^ and/or CD8^+^ T cells from T cell receptor (TCR) transgenic mice, specific for an antigen expressed on the beta cells, enables the tracking of islet-specific cells throughout the body. To our knowledge, there is currently no way of tracking naturally antigen-specific cells in a spontaneous and multi-antigenic model of T1D, such as the NOD mouse. Peptide–MHC multimers are routinely used to find antigen-specific cells by immunofluorescence or flow cytometry, but their use in intravital microscopy is most likely hindered by dose/price, low binding efficiency *in vivo*, and low signal when bound to their specific TCR.

Even though the intravital studies discussed above provided new insights into T cell trafficking to and within the pancreas, it is still to a large extent uncertain what role antigens play in the accumulation of CTLs at the islets. Many of the T cells found at islets in both mouse models (84) and human T1D pancreata (85) are not specific to known islet antigens. Other signals may, thus, be involved in the recruitment of T cells to the islets, such as chemokines and cytokines, and the pathogenic role and behavior of these non-antigen-specific bystander T cells are unknown. Intravital microscopy could provide clues to the behavior and significance of these bystander T cells.

Intravital imaging of the pancreas during onset of T1D also holds the possibility to reveal details on lymphocyte trafficking on a larger scale. Even though they did not perform intravital imaging, Magnuson et al. (86) had an elegant approach to attempt to delineate the whole-body trafficking of antigen-specific and polyclonal lymphocytes in the NOD mouse. By using cells from the transgenic Kaede mouse (87), which carries a photoswitchable protein, the authors could shine an ultraviolet light on cervical lymph nodes and thereby track the color-converted cells throughout the mouse body by flow cytometry. Also, this study found the islets to be “open” to both antigen-specific and non-antigen-specific T cells, as both types trafficked to and from the pancreas to similar extents. The intravital imaging study by Lindsay et al. (55) came to a similar conclusion regarding the governance of antigen over the behavior of T cells. In this study, there was, however, a slight shift in the local behavior of antigen-specific CD4^+^ T cells with time and the progression of islet infiltration. These cells arrested and interacted to a larger extent with antigen-presenting cells at early compared to later stages of disease (55). The study was not able to explain this shift in behavior, but it might be influenced by the major change in microenvironment that the progressive massive infiltration of T cells constitutes. The two studies discussed in this paragraph take either a whole-body- or an islet-focused approach to T cell trafficking. What is lacking in the literature is data on the migration of T cells and antigen-presenting cells from the islets to the pancreatic draining lymph nodes and the importance of this flux of cells. Incorporating a lymphatic stain or a lymphatic genetic fluorescent reporter in intravital pancreas imaging could reveal key steps in early local trafficking events in autoimmune diabetes.

A cell type of great interest in autoimmunity and the focus of many-a-study study in this field is the regulatory T cell (Treg). The potentiation of Treg activity holds the promise of suppressing the immune system in a wide range of diseases (88), and is also being explored in the treatment of patients with recent-onset T1D (89). The T cell trafficking study by Magnuson and colleagues (86) also included Tregs in their analyses. It was found that Tregs were slow in exiting peripheral lymph nodes and slow in migrating to the pancreas, which may explain the decreasing proportion of Tregs in islets as T1D progresses. Tregs are just not repopulating islets in the same rate as conventional T cells are (86). Tregs have been imaged in the pancreatic draining lymph nodes of NOD mice (90), and in islets transplanted to the anterior chamber of the eye of NOD mice (91), but to our knowledge never directly in the living pancreas. The graft study revealed intense interactions between Tregs and effector T cells (91), whereas the lymph node study found much interactions between antigen-presenting cells and Tregs (90). The interactions between Tregs and effector T cells at islet grafts were independent of the local presence of an antigen-presenting cell (91), which makes it even more interesting to see whether there is a similar local immune suppression in the pancreas, and how Tregs behave and migrate in the pancreas.

B lymphocytes have been shown to be involved in both human T1D (92) and mouse models of autoimmune diabetes (93). Even though they are not abundant in the pancreas in the mouse during T1D, their interplay with other infiltrating lymphocytes at the islets has, to our knowledge, not been explored, and may be one of many aspects of the islet immune environment to be interrogated by intravital microscopy.

*In vivo* microscopy of the human pancreas has, to our knowledge, not been performed to date, and is obstructed by the numerous limitations mentioned above that the pancreas imposes on anyone that attempts a surgical procedure on the organ. However, technical progress may in the future lead to high-resolution optical imaging of the islets during T1D onset. A recent report describes a human intravital setup for studying melanomas (94). Imaging the pancreas with a similar setup would require invasive surgery, but the development of more advanced confocal laparoscopes (95) may enable minimally invasive imaging of immune responses. Until then, animal models alongside *ex vivo* preparations of human pancreata (67) provide the best models of studying immune cells at the islets to understand the chain of events during onset of T1D.

## Conclusion

The development of *in vivo* imaging of the pancreas and the immune response during autoimmune diabetes holds great promise for both clinical aspects and preclinical research of T1D. Imaging the beta cell mass non-invasively with great precision in patients with T1D would not only provide a tool for early disease detection and/or detection of persons at risk, but could also provide researchers with invaluable data on the natural course of events of a disease that affects an organ that is very hard to reach for imaging or biopsies.

Imaging islet inflammation in the T1D patient is, however, even more difficult, even if it already has been explored by targeting macrophages. Intravital imaging of murine pancreatic islets *in situ*, transplanted elsewhere, or as explants, has provided the research community with great insights into islet physiology, the immune events during beta cell destruction, and the trafficking of immune cells. The microenvironment that the pancreas holds for the islets and the resident and infiltrating immune cells has repeatedly been shown to have special properties, and should thereby not be neglected when imaging immune responses in T1D.

Much remains to be learnt in this disease, and intravital microscopy is a powerful tool to help answer many questions. With the advent of new microscopy techniques (increased resolution, more channels of data), new genetic reporters (biosensors, photoswitchable proteins), and ways of tissue manipulation (optogenetics, sonogenetics), we may soon be able to get the complete picture of T1D, a picture that currently lacks in resolution.

## Author Contributions

All authors listed have made substantial, direct, and intellectual contribution to the work and approved it for publication.

## Conflict of Interest Statement

MH is an employee of Novo Nordisk. GC declare that the research was conducted in the absence of any commercial or financial relationships that could be construed as a potential conflict of interest.
